# Isolation of human fetal intestinal cells for single-cell RNA sequencing

**DOI:** 10.1016/j.xpro.2021.100890

**Published:** 2021-10-22

**Authors:** David Fawkner-Corbett, Ana Sousa Gerós, Agne Antanaviciute, Alison Simmons

**Affiliations:** 1 Medical Research Council (MRC) Human Immunology Unit, MRC Weatherall Institute of Molecular Medicine (WIMM), John Radcliffe Hospital, University of Oxford, Oxford OX3 9DS, UK; 2 Translational Gastroenterology Unit, John Radcliffe Hospital, Oxford OX3 9DU, UK; 3 Academic Paediatric Surgery Unit (APSU), Nuffield Department of Surgical Sciences, University of Oxford, Oxford OX3 9DU, UK; 4 MRC WIMM Centre For Computational Biology, MRC Weatherall Institute of Molecular Medicine, John Radcliffe Hospital, University of Oxford, Oxford OX3 9DS, UK

**Keywords:** Cell Biology, Cell isolation, Single Cell, Flow Cytometry/Mass Cytometry, Developmental biology

## Abstract

The intestine has a large number of cell types. Thus, digestion of pure and viable populations is necessary for downstream techniques including single-cell RNA sequencing. We outline a protocol to isolate both epithelial and non-epithelial cells from human fetal samples at high viability, which was used to produce a full thickness atlas of intestinal cells across human development. This protocol can also be adapted to adult endoscopy and surgical specimens.

For details on the use of this protocol, please refer to Fawkner-Corbett et al. (2021).

## Highlights

• Outlines preparation and digestion of intestinal tissue for scRNA-seq
• Works with cryopreserved tissue
• Adaptable to different tissue types such as biopsy and surgical tissue
• Can generate samples for multiple downstream protocols

## Graphical abstract

**Figure:**
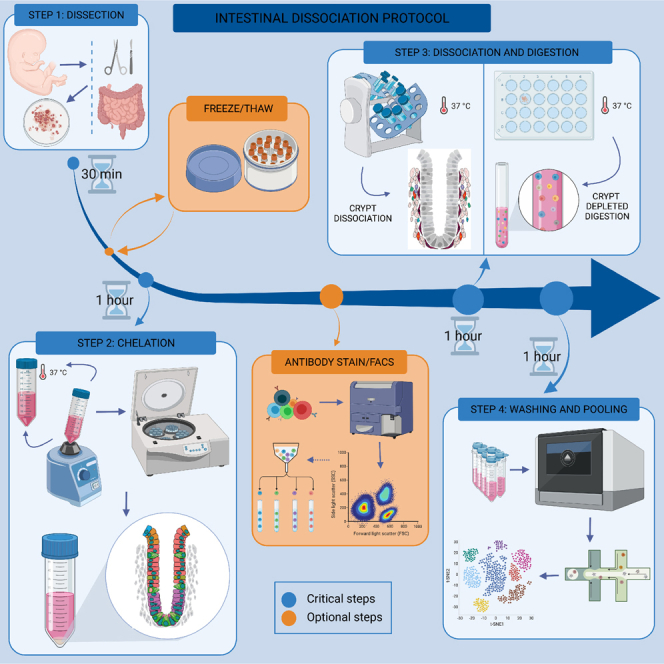

## Before you begin

**Timing: [1.5–3h]**

***Note:*** All tissues should be collected following appropriate ethical and institutional approvals.

1. Prepare the necessary buffers: Transport medium, HPGA, Wash medium, Chelation medium, Thaw medium and Buffer L replacement.
2. Set water bath to 37°C.
3. Set benchtop centrifuges to 4°C.

***Note:*** Set two centrifuges at 4°C. One for both 50 mL and 15 mL tubes, and a smaller one for 1.5 mL vials. All centrifuge steps in this protocol are performed at 300G, 4°C for 4 min.

4. If processing cryo-preserved tissue from liquid nitrogen storage, bring samples to a −80°C freezer for at least 1 h.
5. Thaw a 50 mL aliquot of sterile filtered Fetal Bovine Serum.
6. In a 24-well plate, add 700 μL of Buffer L replacement to one well and 2 mL of Buffer L replacement to another well. Repeat this for each sample being processed. Place the 24-well plate in an incubator set at 37°C.

***Note:*** Protocol example is for fetal intestinal tissue, but can also be performed on adult endoscopic biopsies or surgical tissue with key adjustments mentioned in protocol. Compartment specific adaptations have also been used in adult tissue in (Parikh et al., 2019) and (Kinchen et al., 2018).

## Key resources table

**Table:** 

REAGENT or RESOURCE	SOURCE	IDENTIFIER
**Chemicals, peptides, and recombinant proteins**		

Penicillin-Streptomycin	Sigma	Cat#P0781-100ML
HEPES Buffer Solution (1M)	Gibco	Cat#15630-056
Dulbecco’s Modified Eagle’s Medium (DMEM)	Sigma	Cat#D5796-500ML
HBSS medium	Lonza	Cat#10–543F
Fetal Bovine Serum	Sigma	Cat#F9665-500ML
Ultrapure 0.5M EDTA, pH 8.0	Invitrogen	Cat#15575-038
CryoStorCS10	Sigma	Cat#C2874-100ML
Phosphate-buffered saline (PBS, or similar)	Oxoid Ltd	Cat#BR0014G
ACK Lysis Buffer	Gibco	Cat#A1049201
TrypLE^TM^ Express	Gibco	Cat#12605028
Deoxyribonuclease I from bovine pancreas (DNase)	Sigma	Cat#DN25-10mg
Trypan Blue Stain (0.4%)	Invitrogen	Cat# T10282
DAPI solution (dilution used 1:1500)	BD Pharmingen	Cat#564907
CD90(Thy1) FITC anti-human (dilution used 1:50)	BioLegend	Cat#328107 RRID: AB_893438
CD326(EpCAM) PE-vio770 (dilution used 1:50)	Milteyni Biotec	Cat# 130-099-742 RRID: AB_2660305
CD45 APC human (dilution used 1:75)	Miltenyi Biotec	Cat# 130-098-143 RRID: AB_2660416

**Other**		

Costar 24-well cell culture plates (or similar)	Corning	Cat#3526
Blunt needle	Terumo	Cat#BN-1838
Terumo 5 mL syringe without needle (or similar)	Terumo	Cat#SS∗05SE1
VWR Cell Strainer 70 μm (or similar)	VWR	Cat#732-2758
Pre-Separation Filters 30 μm (or similar)	Miltenyi Biotec	Cat#130-041-407
CoolCell^TM^ LX Freezing Container (or similar)	Corning	Cat#BCS-405G
MACSmix™ Tube Rotator	Miltenyi Biotec	Cat#130-090-753
Countess™ 3 Automated Cell Counter (or similar)	Thermo Fisher	Cat# AMQAX2000

## Materials and equipment

**Table:** Transport medium

Reagent	Final concentration	Amount
DMEM (high glucose)	n/a	490 mL
Penicillin / streptomycin	100 U/mL Penicillin, 0.1 mg/mL Streptomycin	5 mL
HEPES	10 mM	5 mL
Total	**n/a**	**500 mL**

**Table:** HPGA buffer

Reagent	Final concentration	Amount
HBSS	n/a	490 mL
Penicillin / streptomycin	100U/mL Penicillin, 0.1 mg/mL Streptomycin	5 mL
HEPES	10 mM	5 mL
Total	**n/a**	**500 mL**

**Table:** Wash medium

Reagent	Final concentration	Amount
HPGA buffer	n/a	49.9 mL
EDTA (0.5M)	1 mM	100 μL
DTT (powdered)	1 mM	n/a (1 tube)
Total	**n/a**	**50 mL**

**Table:** Chelation media

Reagent	Final concentration	Amount
HPGA buffer	n/a	49 mL
EDTA	5 mM	500 μL
DTT	2 mM	n/a (2 tubes)
FBS	1%	500 μL
Total	**n/a**	**50 mL**

**Table:** Thaw media

Reagent	Final concentration	Amount
Transport Media	n/a	47.5 mL
FBS	5%	2.5 mL
Total	**n/a**	**50 mL**

**Table:** Buffer L replacement

Reagent	Final concentration	Amount
DMEM (high glucose)	n/a	46.25 mL
FBS	5%	2.5 mL
HEPES	25 mM	1.25 mL
Total	**n/a**	**50 mL**

***Note:*** FBS should be sterile filtered through a 0.22 μm filter.

***Note:*** All buffers can be prepared up to 4 weeks before the experiment and stored at 4°C.

**CRITICAL:** Precautions for reagents mentioned above include:

• EDTA causes serious irritation when in contact with the skin and eyes. To avoid this, wear protective gloves/clothing/eye and face protection, and wash skin thoroughly after handling.
• DTT is harmful if swallowed and causes skin/eye/respiratory irritation. It is also harmful for aquatic life with long lasting effects. To avoid this, wear protective gloves/clothing/eye and face protection, and avoid breathing dust/fume/gas/mist/vapors/spray, preferably by handling inside a fume cabinet. Avoid release to the environment.

## Step-by-step method details

### Processing of tissue

**Timing: [<30min]**

Prepare tissue for digestion protocol or storage.

1. Receive intestinal tissue in 10 mL of transport medium on ice and proceed quickly with all dissection steps.

***Note:*** Transport of samples should be performed as quickly as possible but good results in viability are still seen up to 3 h after extraction of intestine from primary sample if kept on ice for the entirety of this process. Timing of dissection should aim to be completed in less than 30 min from starting to freezing or downstream processing, with all reagents pre-cooled on ice.

2. Place tissue on cutting board and examine fetal intestinal tissue under operating microscope. Identify anatomical landmarks for orientation (stomach, appendix, Meckel’s diverticulum) to inform selection of tissue for further processing.
3. Isolate and separate 2 cm regions of tissue from the anatomical compartments of interest (e.g., from terminal ileum, proximal colon and distal colon), or 1 cm in low gestation tissue (e.g., <12 post conceptual weeks [pcw]).
4. Wash lumen with cold PBS to remove meconium. This can be achieved by flushing PBS into the lumen with the help of a blunt needle and syringe or with a pipette tip in larger samples. Wash with 500μL–1000μL depending on size of tissue for at least 3 washes. Late samples (e.g., after 15pcw) may require more to remove visible meconium. Early samples (e.g., <12pcw) may not be large enough to permit a syringe or pipette tip, in which case proceed and the lumen can be cleaned by opening of shaking of the small fragments generated in latter steps.
5. Dissect each sample further by opening the lumen and cutting it into small (e.g., 0.2 cm^2^) tissue fragments.

***Note:*** A similar process can be performed with adult endoscopic biopsies or in adult surgical tissue, with the muscularis mucosae being removed and tissue being dissected into small fragments. In both instances the small fragments will then proceed in a similar manner to that outlined below.

6. Move all tissue fragments to tubes, washing each of them with 10 mL of cold transport media. Shake the tube and centrifuge.
7. [Optional] Tissue can be cryopreserved. For this, fully aspirate all transport media and resuspend the tissue fragments in 500 μL of Cryostor CS10. Place the tubes in a freezing container (e.g., CoolCell® or MrFrosty®) and move it to a −80°C freezer. This will benefit batch processing and multiplexed approaches, and it has shown good recovery of epithelial and non-epithelial fractions even after 6 months.

**CRITICAL:** All dissection steps should be carried on ice using reagents pre-cooled on ice.

### Isolation of epithelial crypts

**Timing: [1h]**

Obtain supernatant enriched for epithelial crypts through EDTA chelation.

8. Prepare tissue for chelation (This step is dependent on the processing method):
  a. If processing fresh tissue:
    i. Centrifuge the tubes containing tissue fragments in transport media.
    ii. Fully aspirate transport media and add 3 mL of ACK lysing buffer. Shake the tube and incubate it at room temperature (18°C–22°C) for 5 min.
    iii. Add 10 mL of wash media and centrifuge.
    iv. Fully aspirate wash media.
    v. Repeat step iii then proceed to Step 9.
  b. If processing frozen tissue:
    i. Per sample, prepare a 50 mL tube containing 25 mL of thaw media using a serological pipette.
    ii. Obtain samples from −80°C freezer and transport them on ice to the water bath.
    iii. Thaw samples in the water bath until only small ice crystal remains (est. 2 min)
    iv. Immediately add thaw media to fill cryovial, mix x3, then transfer contents to the tube containing 25 mL of thaw media.
    v. Agitate the tubes and centrifuge.
    vi. Aspirate thaw media and wash tissue with 10 mL of wash medium. Proceed to step 9.
9. Aspirate final wash media and replace with 10 mL of pre-warmed chelation media.
10. Shake then incubate tubes in a water bath for 15 min. There will be a total of three chelation steps of 15 min each during the epithelial isolation.
11. Vortex or shake tube every 5 min for 5–10 s.
12. After the last vortex, allow tissue to settle. Remove supernatant with FBS-coated pipette and transfer it to a new 15mL tube - crypt fraction 1. Place this tube on ice.

***Note:*** A small amount of chelation media (est. 500 μL can be left in the two initial chelation stages, to preserve tissue remaining for subsequent repetition of chelation steps.

13. Add 5 mL of fresh chelation media to the tissue containing tube, and repeat steps 10–12. In the meantime, centrifuge the tube containing the crypt fraction 1. Resuspend small pellet in 2 mL cold transport medium in a 15 mL tube and place it on ice.
14. Once the second 15 min incubation is complete, repeat steps 12 and 13 to get crypt fraction 2. Pool with crypt fraction 1 and place the tube (now with 4mL transport media) on ice.
15. Repeat the chelation step for a final time by adding 5mL chelation medium and repeating steps 10–12 for crypt fraction 3.
16. In the meantime, during breaks between the EDTA chelation steps, make up stromal digestion enzymes (see below).
17. Repeat steps 12 and 13 (without re-adding chelation medium to the tissue) once again to obtain the third and last crypt fraction. Pool all 3 fractions (6 mL) in a 15 mL tube and centrifuge it.
18. Fully aspirate the all chelation media from the remaining tissue, and then transfer remaining tissue directly to stromal digestion in the incubator.
19. Resuspend all crypt fractions in 3 mL of warm TrypLE Express with 50 μg/mL DNAse, and move the tube into the incubator. Same volume of TrypLE is used for early and late gestation samples.
20. Incubate at 37°C for 35 min using continual agitation or rotation (e.g., MACSmix(Milteyni)).

**CRITICAL:** Time crypt fraction incubation with stromal digestion: move remainder of crypt depleted tissue straight into the stromal digestion cocktail with blow out pipette (Step 18, prepared during chelation steps as detailed below).

### Digestion of crypt-depleted fraction and obtaining single epithelial cells

21. During steps 14–17 of crypt isolate, prepare enzyme solution in the previously pre-warmed 24-well plate containing 700 μL of buffer L replacement.
22. Add the enzymes as follows: 100 μL of Enzyme D, 62.4 μL of Enzyme P, 6 μL of Enzyme B and 12 μL of Enzyme A. Small samples (e.g., <14pcw, or endoscopic biopsies) may need only half volume of each enzyme in cocktail, subjected to optimization.

***Note:*** Enzyme concentrations and timings in this step can be optimized to volume of tissue processed or to time with the epithelial single cell isolation step by altering enzyme of buffer volumes.

23. Place the plate, with crypt depleted fraction added, in the incubator for 45 min, bringing it out every 15 min for mechanical dissociation using a blunt needle and a 2.5 mL syringe.
24. With the needle and syringe, pull up and flush down the sample repeatedly, washing the needle in the 2 mL buffer L replacement well in between uses. Samples may not pass through blunt needle initially.
25. After 45 min – to time with epithelial isolation - look at the sample under a microscope and check for tissue disruption and single cell suspension (Figure 1).

**Figure 1:**
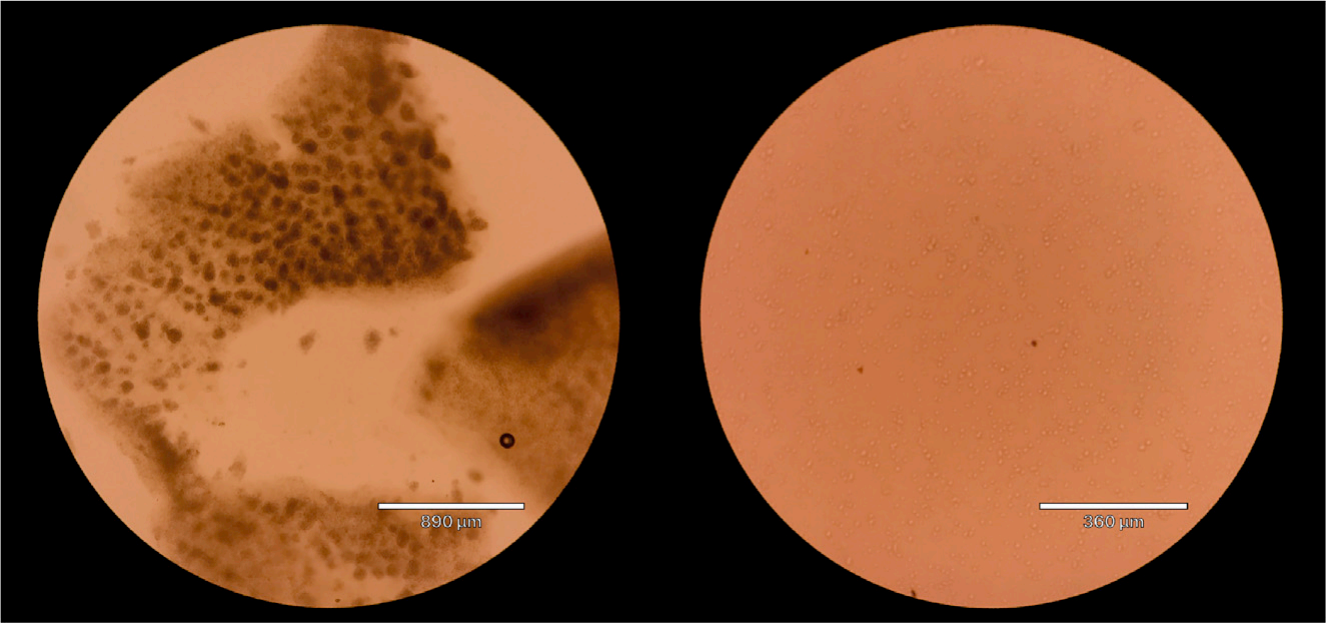
Example of timing of non-epithelial fraction digestion Digestion of the crypt depleted fraction as seen by microscopy at 5 (left) and 40 min (right) in 24 well plate over 40 min. Right image demonstrated good dissociation of single cells at which stage digestion should be stopped (scale bar with respective measurement shown).
26. Collect all stromal and epithelial suspensions. Wash each tube with thaw media and centrifuge. In the meantime, place 70 μm cell strainers on top of 50 mL Falcons – 1 per condition - and pre-wet the filter membrane using FBS.
27. Vigorously resuspend each pellet in 1 mL of thaw media and flush it through the filter. Add thaw media up to 10 mL and centrifuge. Repeat the washing step.
28. Resuspend pellet in 1 mL of thaw media and flush it through a 30 μm strainer pre-wetted with FBS. Wash through with 3 mL of thaw media if using sample for downstream culturing/FACS, or with PBS+ 0.04% BSA for scRNA-seq. Centrifuge.
29. Resuspend cell pellet in 250 μL media or PBS+0.04% BSA depending on downstream application and transfer exactly 250 μL to a new Eppendorf.
30. Take sample for cell counting (e.g., 10 μL). Mix it with 10 μL of 0.1% Trypan Blue and add it to an Invitrogen Countess slide or view under hemocytometer for manual counting of cells and viability.
31. Single cell suspension can then proceed to further downstream use; loading for scRNA-seq on 10× Chromium platform, antibody staining for flow cytometry or scRNA-seq multiplexing or other desired experiments.

**CRITICAL:** Step 28 is optional and can be omitted depending on downstream use of the cells (e.g., in culture or for flow cytometry) but is essential for scRNA-seq.

## Expected outcomes

**Figure 2:**
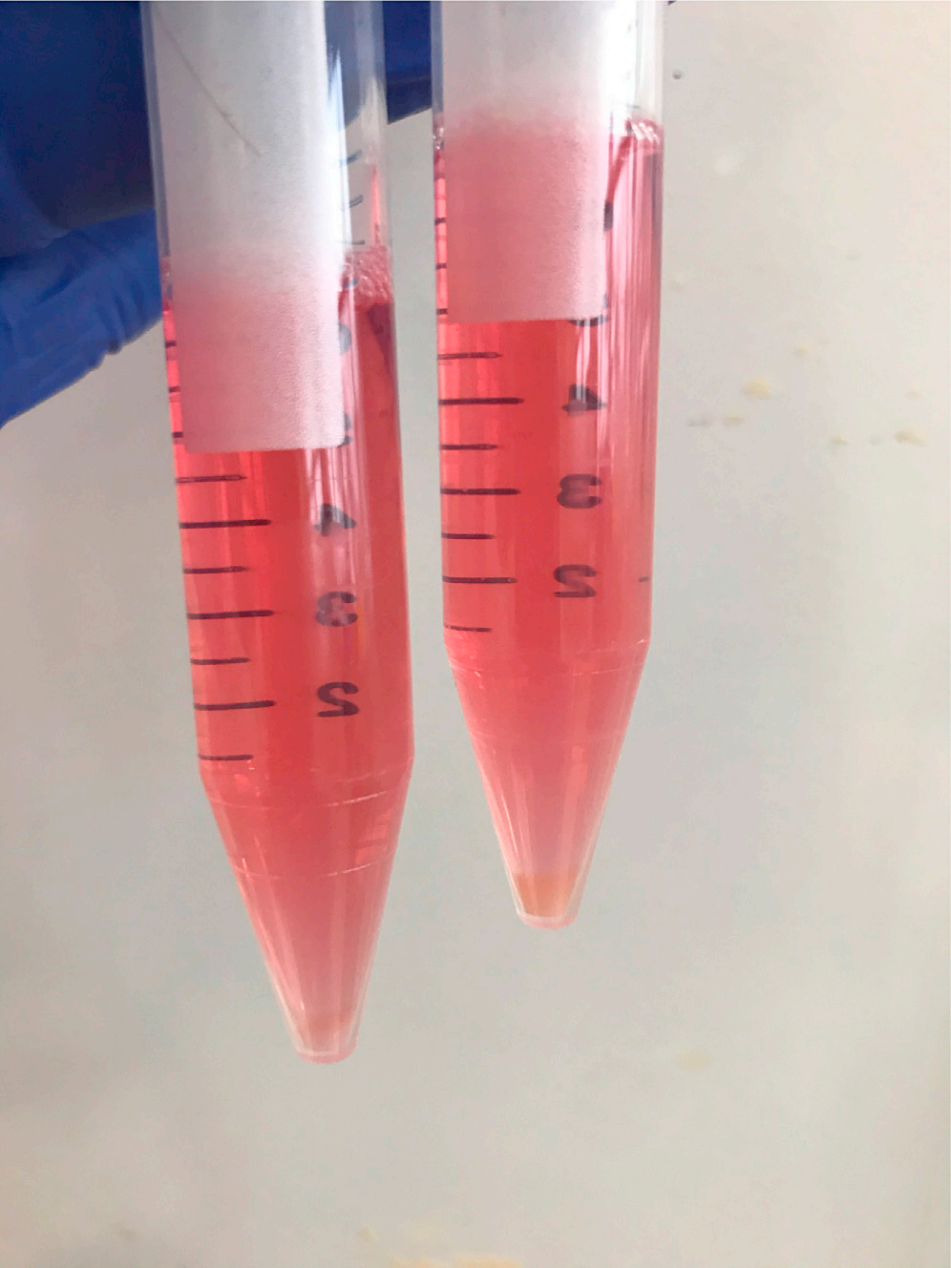
Cell pellet obtained from digestion of both compartments Example cell pellet obtained from the epithelial fraction (left) and crypt-depleted fraction (right) at end of protocol, with less epithelial cells obtained which is expected.

Expected yields of cells can vary depending on the gestational age of sample and the amount of tissue used in the initial dissection step. At initial count after isolation the epithelial depleted fraction is usually of higher cell number and viability, using a hemocytometer or automated cell counter. This can also be visualized in the cell pellet before filtering (Figure 2).

For a recent digestion of n=25 samples from 8–22 post conceptual weeks, viabilities and total cell counts were assessed using a Countess II (ThermoFisher, example Figure 3.1a) both straight after the digestion protocol and then following a 30-min antibody stain to attach hashtag oligonucleotide antibodies with associated washes before undergoing multiplexed scRNA-seq (Fawkner-Corbett et al., 2021; Stoeckius et al., 2018). There is a significant drop in recovery and viability during the staining process, but the majority had viabilities >70% for epithelial and >80% for non-epithelial and were able to be run on the 10× Genomics platform with good result (Epithelial fraction: Mean recovery of viable cells after stain 230,122 cells (S.E.M 61,766) and viability 75.04% (S.E.M. 2.13%); non-epithelial fraction: Mean recovery of viable cells 555,938 cells (S.E.M. 79,324) and viability 81.92% (S.E.M. 1.95%) Figure 3.1b).

**Figure 3:**
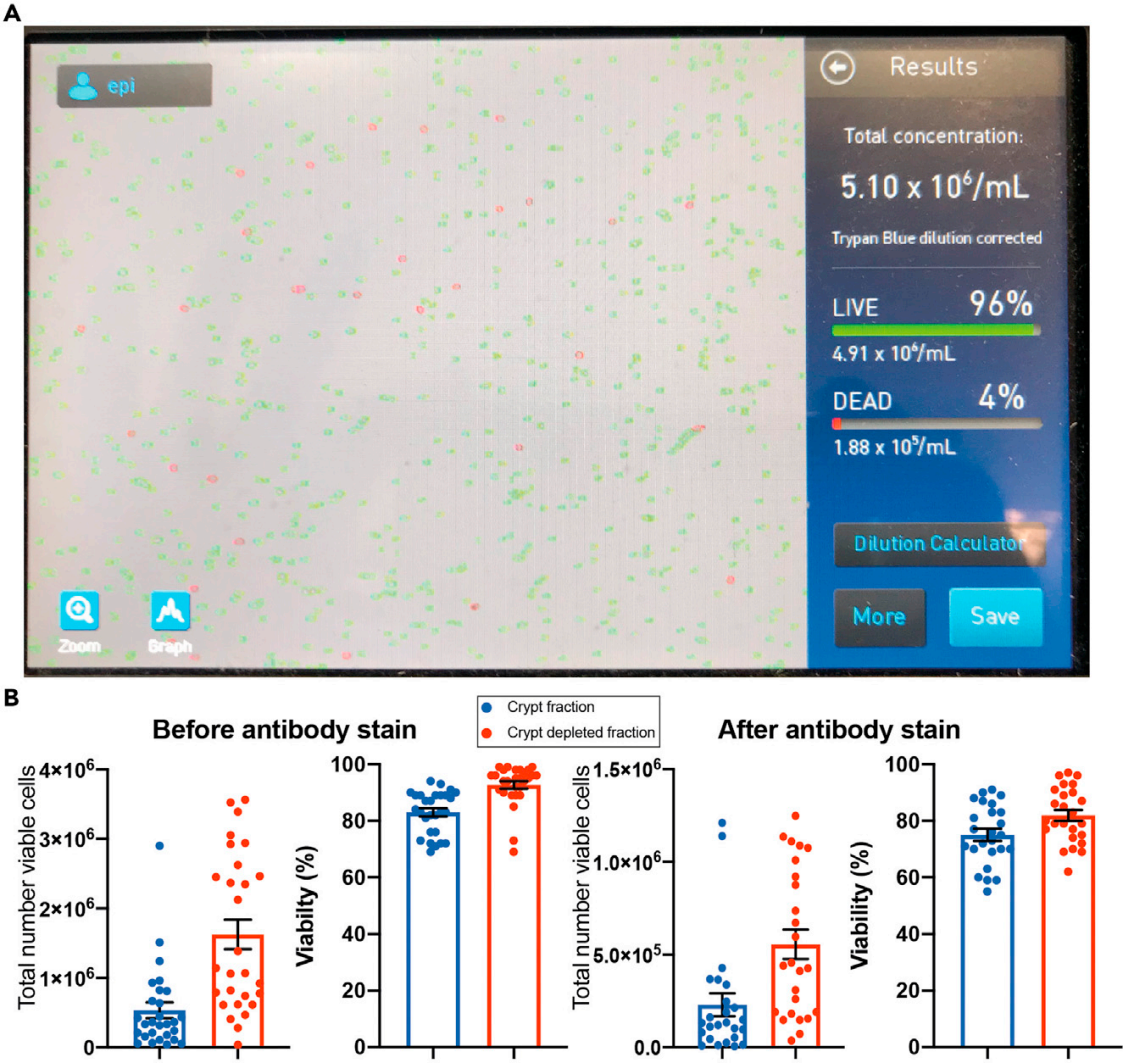
Assessment of final digested single cell product (A) Example image from countess II (ThermoFisher) with non-epithelial fraction in single cell suspension showing good viability and dissociation (Dilution volume 250 μL, count profile set previously with hemocytometer). (B) Overview of total number of viable cells and percentage viability from immediately after digestion and after hashtag oligo antibody staining protocol (Stoeckius et al., 2018) in n=25 samples of fetal intestine (Error bar representing mean +- standard error of the mean (SEM)).

**Figure 4:**
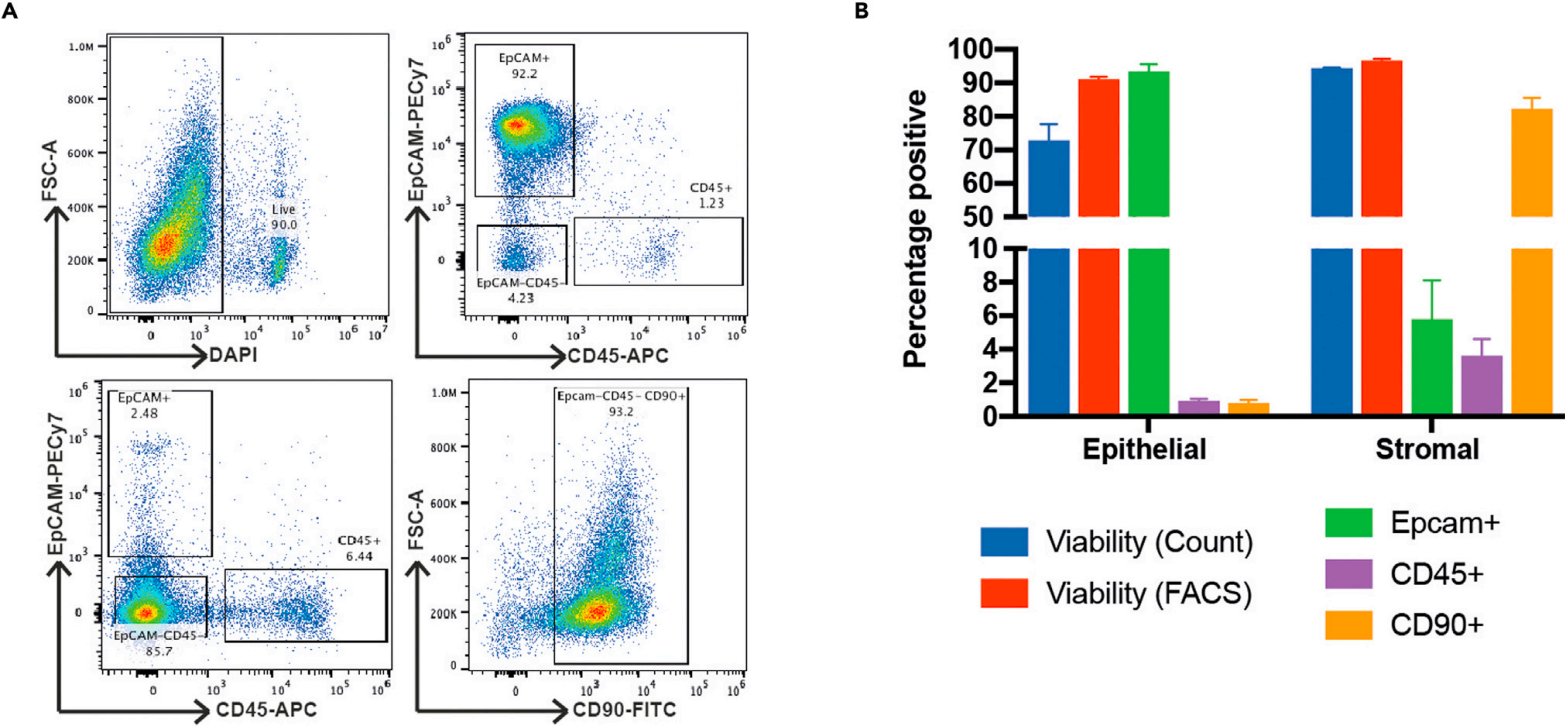
Purity of digestion fractions assessed by flow cytometry (A) Example gating strategy to quantify singlets (SSC-A vs FSC-A, not shown) which were viable (DAPI-) and identified as in epithelial (EpCAM+CD45-), immune (EPCAM-CD45+) or stromal (EPCAM-CD45-) compartments and quantified (B). (Epithelial digestion fraction top, non-epithelial digestion fraction bottom, n=3 repeats, error bar representing mean +SEM)

Flow cytometry has also demonstrated that the enriched populations are highly purified, with only a small percentage of stromal cells (EPCAM-, CD90+) being identified in the crypt population and epithelial cells (EPCAM+) in the crypt depleted proportion (Figure 4). Similar results have been shown from healthy biopsied taken from adults during endoscopy although the proportion of immune cells (CD45+) can be much greater in both of the digested fractions, especially in the context of IBD associated inflammation (Data not shown).

This protocol is designed to recover both the epithelial and non-epithelial populations containing all cell types in the full thickness of intestine. Further alterations can be performed if targeting one cell type of interest. This could include undertaking only the first part of protocol to obtain epithelial cells(Parikh et al., 2019).Alternatively, to obtain an enriched intestinal fibroblast population, the second enzyme digestion could be performed on the whole tissue, followed by bead separation of epithelial (CD326), immune (CD45) and erythrocyte (CD235a) populations using antibody conjugated beads (Miltenyi) as per(Kinchen et al., 2018). A similar method could select immune (CD45) cells by positive selection, or flow cytometry to sort a population of interest.

The epithelial fraction can be cultured to form epithelial organoids by placing the isolated cells into Matrigel in line with previous publications (Sato et al., 2011). The antibiotics contained in the media mean that, in our experience, these will culture without infection.

## Limitations

The number of cells obtained is dependent of the amount of tissue dissociated. This protocol has been validated on low gestation samples (e.g., 8pcw) with minimal intestinal tissue, as well as on 2 endoscopic biopsies. In these cases of low tissue input the final yield of epithelial cells is expected to be lower than the crypt-depleted fraction, but epithelial cells should still be obtained. Reduction of dilution volume to 100 μL in the penultimate step of “Digestion of crypt depleted fraction and obtaining single epithelial cells” (step 29) is advised.

A final viability check should be performed to ensure high viability (e.g., >70% in epithelium and >80% in non-epithelial fraction, although at discretion of user) before proceeding to scRNA-seq.

## Troubleshooting

### Problem 1

When counting cells, or looking at them under microscope after digestion, there is a large amount of doublets and cell clumps in the sample (Identified at Step 30 of section “Digestion of crypt depleted fraction and obtaining single epithelial cells”).

### Potential solution

This is most commonly seen in the epithelial fraction, and can be solved by extending the TrypLE digestion for 5–10 more minutes and/or by adding an extra wash and filtering step to the end of the protocol. If necessary, adding 200 μL of buffer L replacement to the non-epithelial digestion can extend the umbilical cord kit (Milteyni) digestion and help account for the additional time required by epithelial fraction so that digestions of both fractions are completed together. This must be balanced with viability of final sample however.

### Problem 2

The viability of cells in the final digested tissue sample appears to be too low. (Identified at Step 30 of section “Digestion of crypt depleted fraction and obtaining single epithelial cells”)

### Potential solution

**Figure 5:**
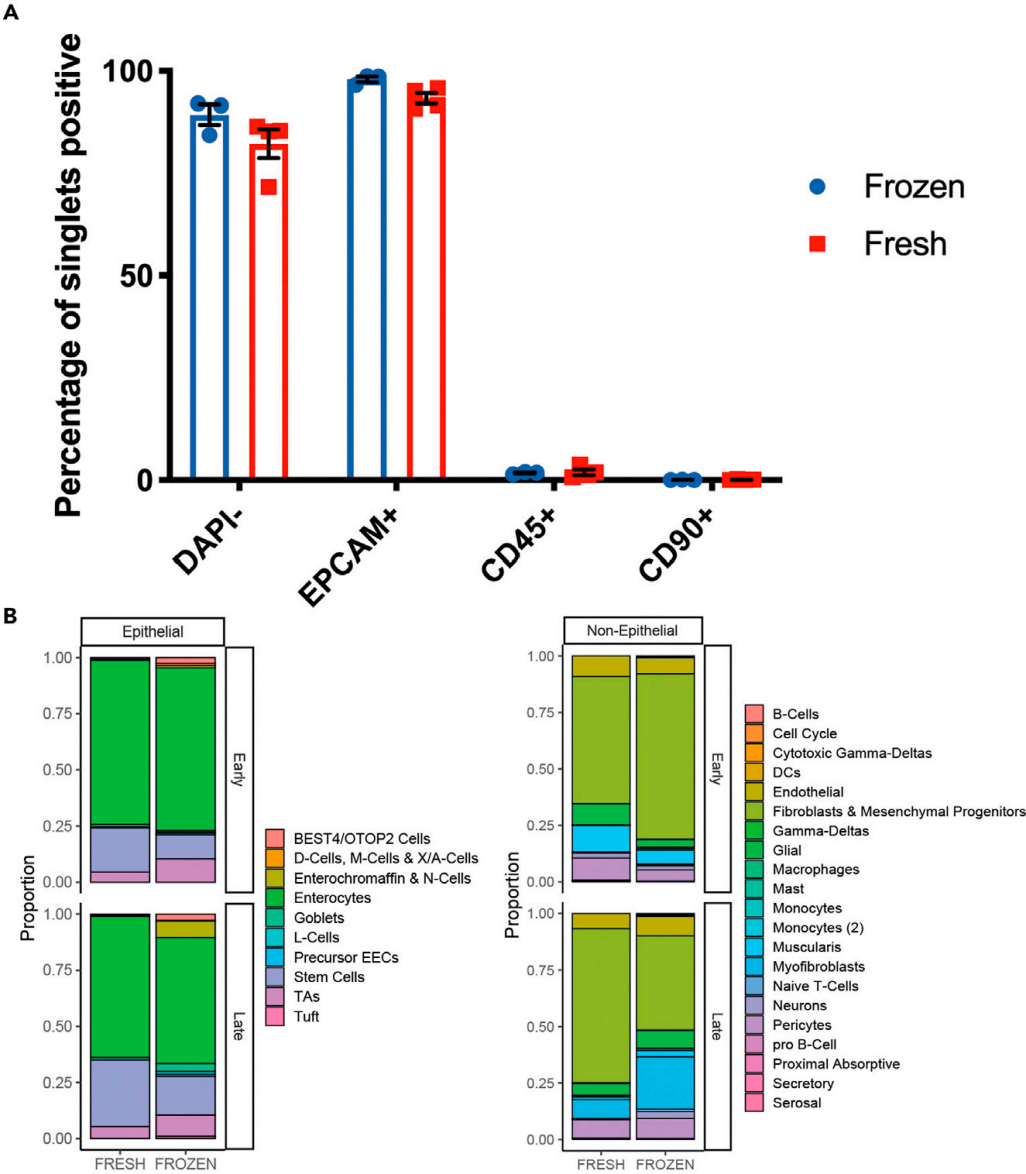
Comparable purity of cryopreserved cells (A) Flow cytometry was performed on epithelial digestion fraction as per example gating strategy in Figure 4A and cell populations quantified (n=3 frozen samples, n=4 fresh tissue from same tissue section, error bar representing mean +-SEM). (B) In a scRNA-seq pilot run fresh and cryopreserved (x-axis) samples from early (12pcw) and late (19pcw, y-axis) intestine were run together with comparison of cell populations (Data represents samples re-analyzed from Fawkner-Corbett et al., 2021).

Again, this is more often seen in the epithelial fraction. Proceeding promptly through the protocol and reducing dissection time before starting the protocol is critical. Transporting and dissecting samples on ice is important. For fetal tissue it was found that the majority of samples will achieve a viability of >70% for epithelial fraction and >80% for non-epithelial (Figure 3). Viability can be further improved by use of live/dead bead separation kit (Miltenyi) or by flow cytometry sorting on a viability dye (DAPI or Zombie fixable violet) but has the limitation of a lower cell recovery. Use of frozen or cryopreserved samples thawed as described in this protocol has shown to have no significant effect on final viability, and has been validated in the more delicate epithelial fraction by flow cytometry and in pilot runs of scRNA-seq to compare cell type proportions (Figure 5 and Fawkner-Corbett et al., 2021).

### Problem 3

The total/live number of cells seems erroneous (Identified at Step 30 of section “Digestion of crypt depleted fraction and obtaining single epithelial cells”).

### Potential solution

To add confidence at final cell counting, it is optimum to repeat it twice, especially when exact enumeration is vital such as when loading the cells directly in single cell protocols. In instances where duplicate counts are vastly different, this is often due to low cell recovery and most often seen in low gestational samples or after antibody staining steps. The final dilution can be changed to 100 μL to improve cell counting accuracy. In addition, if using an automated cell counter, which are often optimized to quantify peripheral blood mononuclear cells, calibration of the pre-set settings with a manual hemocytometer can help improve speed and accuracy.

### Problem 4

Large amount of mucus (seen on microscope or next to pellet) in the epithelial digestion (Identified by eye or looking at crypts under microscope after step 17 of section “Isolation of epithelial crypts”).

### Potential solution

Sometimes mucus can be seen in the epithelial crypt fraction as a light, “fluffy” substance above the pellet either macroscopically, under the microscope when checking digestion or when counting. This appears to have no effect on the digestion process and it is lost in filtering steps. However, it can give the impression of losing the cell pellet. This can be removed by increasing the concentration of DTT in the chelation media.

### Problem 5

My cell type of interest does not appear using cryopreserved tissue (Identified by staining or by analysis at end of protocol)

### Potential solution

It is our experience that use of cryopserved tissue gives the full representation of cell types across the thickness of intestine with comparable mRNA signatures to previous freshly processed samples and allows for multiplexing of samples to increase throughput. This was of use for charting all cell types across developmental time. It is feasible that some cell types may be enriched or depleted with this method, and the cost of scRNA-seq means that direct comparisons of both are challenging although an example is given (Figure 5). If there is concern that cryopreservation depletes a cell type of interest, or reduces the response of a cell type to stimulation afterward then the protocol can still be used to process samples fresh as detailed in section “processing of tissue”.

## Resource availability

### Lead contact

Further information and requests should be directed to and will be fulfilled by the lead contact, David Fawkner-Corbett (David.fawkner-corbett@imm.ox.ac.uk)

### Materials availability

This study did not generate any unique reagents.

## Data Availability

This protocol did not in itself generate any data or code availability. A recent example of data generated from this technique for scRNA-seq is publicly available (Fawkner-Corbett et al., 2021) and deposited on GEO (GEO: GSE158702).
